# The Burden of Adverse Drug Reactions in Africa in the Context of Pharmacogenetics‐Based Clinical Guidelines

**DOI:** 10.1002/cpt.70269

**Published:** 2026-04-03

**Authors:** Janine Scholefield, Tinashe A. Mazhindu, Mohamed Nagy, David Twesigomwe, Gaye Agesa, Collen Masimirembwa

**Affiliations:** ^1^ Bioengineering and Integrated Genomics Group Council for Scientific and Industrial Research Pretoria South Africa; ^2^ Faculty of Health Sciences, Division of Human Genetics, National Health Laboratory Service, and School of Pathology University of the Witwatersrand Johannesburg South Africa; ^3^ African Institute of Biomedical Science and Technology Harare Zimbabwe; ^4^ Faculty of Medicine & Health Sciences, Department of Oncology, Medical Physics and Imaging Sciences University of Zimbabwe Harare Zimbabwe; ^5^ Department of Pharmaceutical Services and Sciences Children's Cancer Hospital Cairo Egypt; ^6^ Personalized Medication Management Unit Children's Cancer Hospital Cairo Egypt; ^7^ Faculty of Health Sciences, Sydney Brenner Institute for Molecular Bioscience University of the Witwatersrand Johannesburg South Africa; ^8^ Medical Research Council/Uganda Virus Research Institute and London School of Hygiene and Tropical Medicine Uganda Research Unit Entebbe Uganda; ^9^ African Population and Health Research Center Nairobi Kenya

## Abstract

Genetic variation has a significant impact on patients' response to medicines. Variations in important pharmacogenes have been evaluated in several studies and consequently led to international guidelines from clinical pharmacogenomics consortia. However, there are limited examples of these being implemented across the African continent despite the increased evidence of how these variants contribute to adverse drug reactions (ADRs) or ineffective treatments. Considering the vast genetic diversity in Africa, there are significant gaps in understanding how much of a negative effect this might be having across healthcare across the continent. We therefore sought to establish the extent of ADRs from a subset of medicines shown to be associated with pharmacogenetic biomarkers from the last 10 years across 47 countries on the African continent. In the absence of pharmacogenetic testing on the African continent, we used international guidelines to derive a subset of definitions associated with published drug‐gene‐interaction associated ADRs to infer the role of pharmacogenetic variation on the prevalence of ADRs. The data showed that African countries report only 1% of ADRs in the VigiBase database, indicative of weak pharmacovigilance programs on the continent. Our analysis revealed that ADRs were mainly caused by anti‐infectives such as efavirenz. The inferred pharmacogenes associated with high prevalence of ADRs were *CYP2B6*, *CYP2D6*, and *CYP2C19*. Using limited data, this foundational analysis may serve as the basis for stakeholders to prioritize pharmacogenetic interventions across the African continent.

**Study Highlights**

**WHAT IS THE CURRENT KNOWLEDGE ON THE TOPIC?**

There is limited knowledge on the extent to which Africa's diverse populations are negatively affected by medicines as a consequence of pharmacogenetic variants.

**WHAT QUESTION DID THIS STUDY ADDRESS?**

This study sought to create a baseline by taking advantage of existing VigiBase adverse drug reaction data collected over the last 10 years from the continent, evaluating medicines associated with pharmacogenetic biomarkers.

**WHAT DOES THIS STUDY ADD TO OUR KNOWLEDGE?**

Despite the lack of direct causal links, in the absence of genetic testing, the data reveal which medicines are potentially causing adverse drug reactions, through drug‐gene interactions, and provide a high‐level view of gaps in reporting in comparison to the Global North.

**HOW MIGHT THIS CHANGE CLINICAL PHARMACOLOGY OR TRANSLATIONAL SCIENCE?**

Our results provide a mechanism to make recommendations for evidence‐based interventions ranging from guidelines for pharmacogenetic testing through to drug‐gene interaction studies and improved drug labelling. Critically, these decisions can take local priorities (from disease burden, drug accessibility, genetic diversity) into account, significantly empowering stakeholders across the continent.

Adverse drug reactions (ADRs) pose a significant threat to the optimal treatment outcomes of populations in the absence of diverse clinical trials. Africa represents 18% of the global population, yet only 3% of clinical trials take place on the continent.^1^ In addition, Africa has a unique disease burden and furthermore constitutes a population of individuals with more genetic diversity than other global biogeographical populations.

Depending on the seriousness of the ADR, the negative impact on the patient in terms of their health outcome, and the increased chances of unplanned hospital stays can present a significant burden on the individual and hospital services in terms of resources. These effects can be life‐threatening, and impact every disease class. Examples include central nervous system (CNS) disorders due to efavirenz toxicity,^2^ haemolytic anemia resulting from malaria treatments,^3^ and peripheral neuropathy caused by anti‐TB medicine.^4^ It is important to understand that while most ADRs are reported due to clinical manifestations (e.g., toxicity) recognized by a healthcare practitioner, many adverse events are ‘unseen’, since ineffective treatment does not necessarily lead to visible side effects. These include commonly prescribed chemotherapeutics such as tamoxifen for which disease recurrence has been reported due to ineffective bioconversion^5^ and the numerous reports of inefficacy from the use of selective serotonin reuptake inhibitors (SSRIs) such as escitalopram.^6^

There is yet to be a consensus on how to evaluate the costs of ADRs, but a 2024 global systematic review of 20 studies (including one African study from South Africa)^7^ indicates significant variability in the range of EUR 6 k–10 k per hospitalization. Given that one in 12 individuals have been reported to be admitted to hospital in South Africa,^8^ this poses a significant financial burden on any state in addition to the impact on the individuals being served.

ADR reports submitted by African countries to the Uppsala Monitoring Centre (UMC),^9^ reveal a reporting rate of three ADRs per million inhabitants in comparison to 130 per million in developed countries from 2000 to 2009,^10^ providing an important perspective of global vs. African reporting. This demonstrates the difficulties in spontaneous reporting by healthcare practitioners—particularly given Africa's resource limited settings. These encompass issues of a lack of pharmacovigilance knowledge across healthcare practitioners, infrastructure available towards accredited reporting instruments (only six African countries hold a maturity level (ML) 3 designation across vaccines and/or medicines^11^), and a strong need for harmonization across the continent, or at least, regional blocs^12^ to support regional prioritization of drug approval.

To better understand the cause and prevalence African ADRs, it is imperative to understand contributing factors that are unique to the region, including disease burden, drug accessibility and the diversity of African genetic variations in pharmacogenes which may provide insight into those drugs/drug classes responsible for ADRs in Africa. African genomic heterogeneity contributes to variable treatment outcomes observed across the continent.^13^, ^14^, ^15^, ^16^, ^17^, ^18^ There is now significant evidence that many ADRs are inherently linked to specific drug gene interactions (DGIs). The most important pharmacogenes responsible for ADRs and sub‐optimal treatments are summarized by ClinPGx (formerly PharmGKB) in different tiers based on collated evidence of support.^19^ Such evidence is also forms the basis of the Clinical Pharmacogenetics Implementation Consortium (CPIC) guidelines.^20^ CPIC publishes updated recommendations that seek to support clinicians in making decisions on genotype–phenotype correlations of DGIs. Similar guidelines exist from the Dutch Pharmacogenetics Working Group (DPWG^21^), the Canadian Pharmacogenomics Network for Drug Safety (CPNDS^22^), and the French National Network (Réseau) of Pharmacogenetics (RNPGx^23^).

Whilst a number of studies have shown the link between specific DGI‐associated ADRs in relation to individual drugs, there is limited knowledge on the extent of DGI‐associated ADRs across Africa as a whole. Towards addressing this knowledge gap, we analyzed the ADRs submitted to VigiBase by African countries between 2014 and 2023 in the context of 56 drugs for which evidence of contributing pharmacogenetic (PGx) factors had been evaluated and confirmed by one or more of the aforementioned working groups and consortia in clinical PGx. Over 40,000 reports were submitted to VigiBase over 10 years for this subset of drugs, which represent only 1% of corresponding global VigiBase reports with the same criteria. Whilst PGx data directly associated with each report is not available, we analyzed a subset of ADRs for each drug, for which global clinical evidence has shown a direct link to variants in a specific gene. Whilst this analysis is limited to associations and cannot be proven to be directly causal, several known global PGx associations are revealed from our VigiBase dataset, and could be used as a foundation on which to base priority PGx studies across the continent.

## METHODS

### Selection of 56 medicines associated with PGx associated biomarkers

Using the international supporting database ClinPGx, (accessed on 15 January 2025), the following list of 56 drugs with defined DGIs were identified from over 500 medicines listed on the 23rd WHO Model Essential Medicines List (EML). This list of 56 drugs is referred to as the Medicines Associated with PGx Biomarkers (MAPBs) dataset:

Abacavir,^24^, ^25^ allopurinol,^26^, ^27^ amikacin,^28^ amitriptyline,^29^, ^30^ aripiprazole,^31^ atazanavir,^32^ atorvastatin,^33^, ^34^ azathioprine,^35^, ^36^ capecitabine,^37^, ^38^ carbamazepine,^39^ cisplatin,^40^ citalopram,^41^, ^42^ clomipramine,^29^, ^30^ clopidogrel,^43^ codeine,^44^, ^45^ dapsone,^46^, ^47^ daunorubicin,^48^ doxorubicin,^48^ efavirenz,^49^ escitalopram,^41^, ^42^ flucytosine,^50^ fluorouracil,^37^, ^38^ fluvastatin,^33^, ^34^ fluvoxamine,^41^, ^42^ gentamicin,^28^ haloperidol,^31^ halothane,^51^, ^52^ ibuprofen,^53^ irinotecan,^54^, ^55^ isoflurane,^51^, ^52^ kanamycin,^28^ lamotrigine,^56^, ^57^ lovastatin,^33^, ^34^ mercaptopurine,^35^, ^36^ metoprolol,^58^, ^59^ nitrofurantoin,^47^, ^60^ omeprazole,^61^, ^62^ ondansetron,^63^ paromomycin,^28^ paroxetine,^41^, ^42^ phenytoin,^64^ plazomicin,^28^ pravastatin,^33^, ^34^ primaquine,^47^, ^65^ rasburicase,^47^, ^66^ ribavirin,^67^, ^68^ risperidone,^31^, ^69^ sertraline,^41^, ^42^ sevoflurane,^51^ simvastatin,^33^, ^34^ tacrolimus,^70^ tamoxifen,^5^ tramadol,^45^, ^71^ tropisetron,^63^ voriconazole,^72^ and warfarin.^73^

### Data extracted from VigiBase

Of the 56 drugs, the following was requested from VigiBase:

- Scope of substance search: only single ingredient
- Drug involvement: Suspected/interacting/concomitant
- Any/ all MedDRA terms:
- Timeline: ADRs in Africa by year from January 2014 to December 2023 were selected to avoid definition variations that occurred prior to 2013
- De‐duplication algorithms were used yielding a report entitled Preferred ICSR where duplicated reports have been removed.

Upon receiving the VigiBase ADR dataset, no reports for plazomicin or tropisetron were recorded. Subsequent analysis was then performed on the remaining 54 drugs.

### DGI associated ADRs

Tight definitions using MedDRA terms linked to evidence‐based datasets (e.g., ClinPGx, CPIC, DPWG, CPNDS and RNPGx) were used to define a subset of DGI associated ADRS linked to specific genes (**Table** **S1**). All assigned terms were linked to specific ADRs shown to be linked to a specific DGI and referenced accordingly as above. Specifically for each drug listed above, the corresponding CPIC, DPWG, CPNDS and/or RNPGx associated evidenced based DGI publications were evaluated. Using these clinically relevant genotype associated ADR guidelines, a set of Medical Dictionary for Regulatory Activities (MedDRA) terms specifically identified in each of these publications, confirmed through evaluations of multiple studies to be associated with variants in a specific gene were considered as a possible subset of total ADRs, i.e., DGI associated ADRs. Once collated for each drug, each summary drug report was assessed, and the MedDRA terms associated with each DGI associated ADRs were counted. This is in contrast to the term “total ADRs” in which our analysis refers to all the MedDRA terms contained in reports submitted to VigiBase associated with a specific drug.

### Ethics

Ethics approval was not required due to the use of public domain data.

## RESULTS

### Assessment of total global ADRs linked to the MAPB dataset

**Figure 1:**
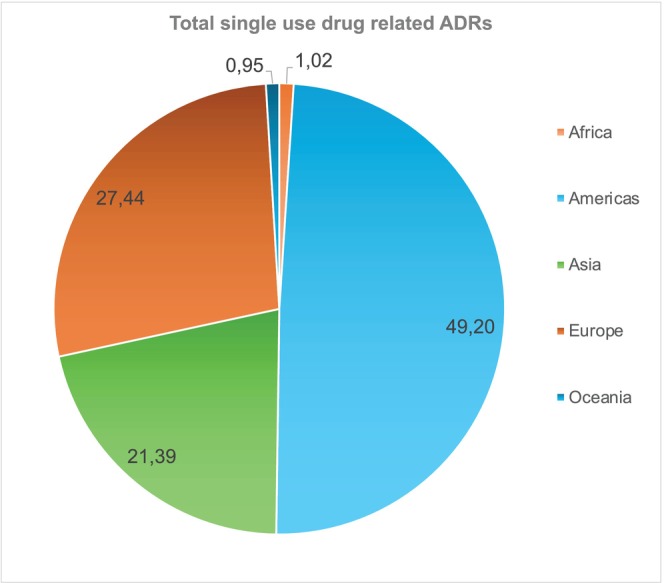
Percentage of single use drug related ADRs per geographical region of the 54 Medicines Associated with Pharmacogenetic Biomarkers (MAPB).

Data extracted from VigiBase revealed that the 54 MAPBs were associated with a total of 4,577,108 reports across the globe representing 7% of all WHO EML ADRs reported over this time period. Across the five regions, Africa and Oceania represented the lowest number of ADR reports constituting 1.02% and 0.95% respectively. The data from Africa represents a total number of 46,728 reports, from 47 countries (**Figure** **1**), which constitute 12% of the ADRs reports from all WHO EML medicines.

### Assessment of MAPB ADRs from Africa

**Figure 2:**
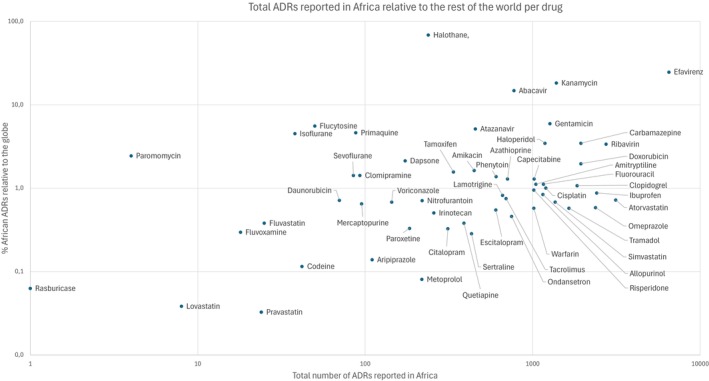
Total ADRs reported in Africa relative to the rest of the world for each medicine. Both axes are shown using a log_10_ scale. The x axis represents the total number of reports for these drugs in Africa, whilst the y axis represents ADRs reported in Africa as a % compared to the rest of the world for each medicine. All dots on the scatter plot are associated with the named MAPB to the right, unless linked by a line.

The data was further interrogated to establish contributions to total ADRs in Africa to assess the impact of each MAPB both in terms of incidence, as well as its impact relative to the rest of the globe (**Figure** **2**, **Table** **S2**). The top 10 medicines most frequently reported as contributing to ADRs across the continent are efavirenz, (6511), atorvastatin, (3134), ribavirin, (2746), ibuprofen, (2410), omeprazole, (2369), doxorubicin, (1941), carbamazepine, (1937), clopidogrel, (1842), tramadol, (1647) and kanamycin, (1387). On the other hand, the top 10 medicines responsible for the highest % ADRs coming from Africa are halothane, (68.99%), efavirenz, (24.84%), kanamycin, (18.28%) abacavir, (14.88%), gentamicin, (5.97%), flucytosine, (5.59%), atazanavir, (5.15%), isoflurane, (4.52%), carbamazepine, (3.49%), and haloperidol (3.48%). Of interest is that three drugs share a position on both lists i.e., efavirenz (treatment of HIV), kanamycin (multi‐drug resistant TB) and carbamazepine (seizures and bipolar disorder).

### Assessment of DGI associated ADRs in Africa

Since many ADRs are related to adverse effects due to inappropriate drug label use, incorrect dosage and other expected side effects, the reported ADRs for each MAPB were further analyzed to only include those MedDRA terms for which clinical evidence has previously shown direct links to PGx variants, and therefore, may be causative of increased toxicity or inefficacy (detailed in **Table** **S1**) as a subset of clinical phenotypes. In the absence of causal genetic data associated with these ADRs, we created a defined subset of ADRs using publications citing extensive clinical data of specific ADRs associated with variants in known pharmacogenes – referred to as DGI associated ADRs.

Based on these refined definitions, contributions of MAPBs to the DGI‐associated ADRs were assessed (**Table** **S3**). The following drugs were associated with the highest numbers of reports from VigiBase (DGI associated ADRs as a combination of those contributing to toxicity and inefficacy): efavirenz (2506), carbamazepine (1353), capecitabine (740), haloperidol (658), kanamycin (612), risperidone (483), fluorouracil (478), atorvastatin (359), warfarin (349), and amitriptyline (297). No DGI associated ADRs, as defined in the methods, were reported for daunorubicin, flucytosine, paromomycin, and sevoflurane.

**Figure 3:**
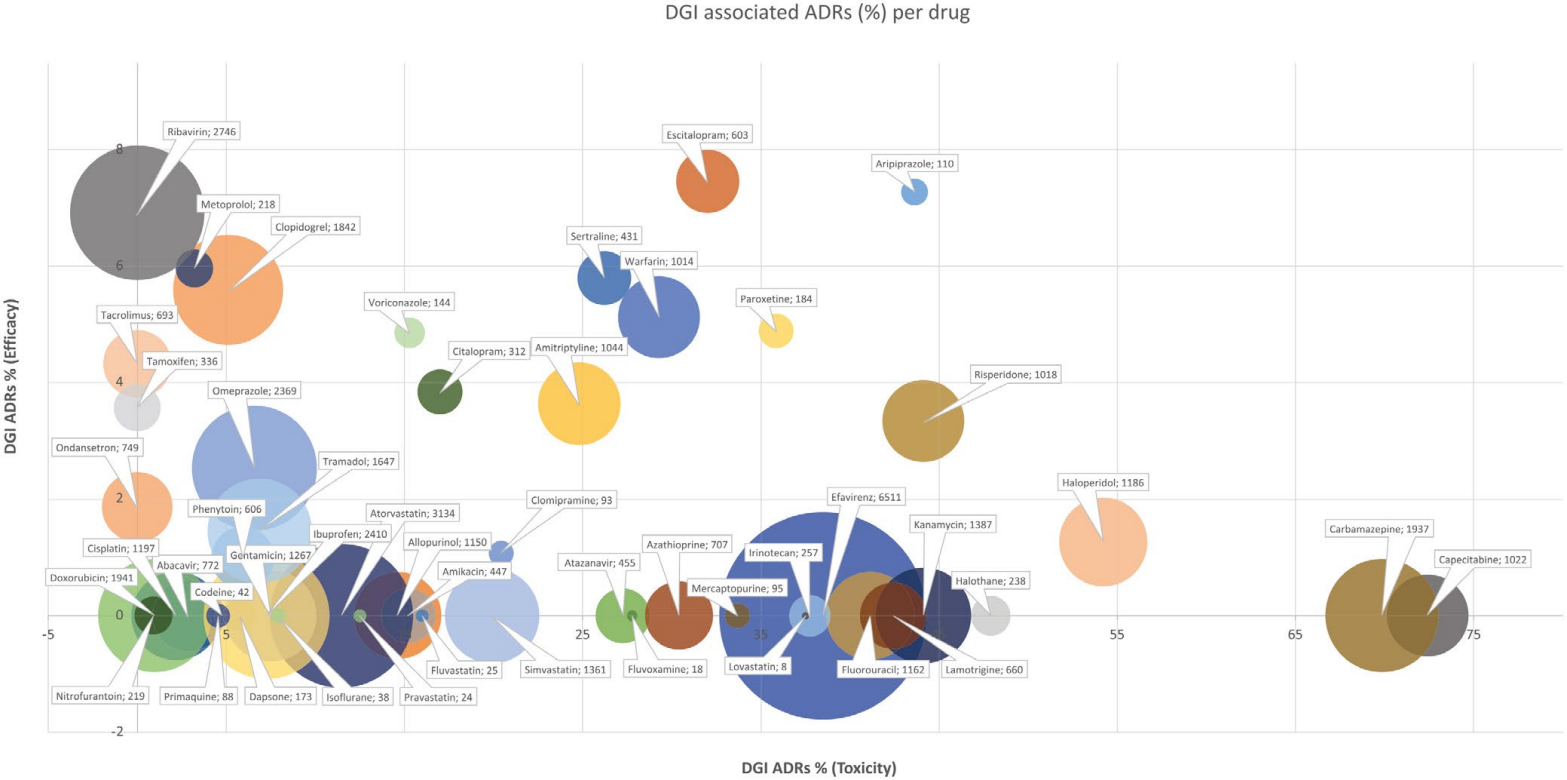
Relative impact of pharmacogenetic variation on ADRs for each MAPB as shown by inferred DGI associated ADRs (%). Each bubble represents the % of potential DGI associated ADRs linked to toxicity (x‐axis) and efficacy (y‐axis) in relation to the total number of ADRs associated with each MAPB. In addition, the number of total ADRs reported per MAPB is represented by the size of the bubble, with the named medicine and the number of inferred DGI associated ADRs as a label. Rasburicase was removed due to the fact that only one ADR was reported, which was linked to a single DGI associated ADR.

In order to understand the potential impact of the relative contribution of DGI associated ADRs from both the point of view of more clinically obvious toxicity related side effects and ineffective treatment, an assessment was made to reveal the distinction between both aspects of DGI associated ADRs relative to total ADRs of each MAPB to the continent. In addition, the analysis was overlaid in a bubble graph to reveal the potential extent of the impact of genetic variants on ADRs across the African continent albeit in the absence of directly causal PGx data (**Figure** **3**).

The analysis reveals the extent of potential PGx contributions to ADRs within the context of the *size* of each report cluster of each MAPB as reported as a percentage of ineffective treatment and toxic side effects, predicted by aforementioned guidelines. The top 10 medicines associated with redefined DGI associated ADR MedDRA terms are capecitabine (72.4%), carbamazepine (69.9%), haloperidol (55.5%), aripiprazole (50.9%), halothane (47.9%), risperidone (47.4%), kanamycin (44.1%), lamotrigine (42.4%), fluorouracil (41.1%), and paroxetine (40.8%).

When assessing those medicines for which VigiBase reports revealed the highest proportion of toxic DGI associated ADRs, this list then includes efavirenz (38.5%), which replaces paroxetine. In contrast, whilst the contributing percentages of DGI associated ADRs listed as ineffective, are significantly lower, the top 10 medicines associated with infectivity are escitalopram (7.5%), aripiprazole (7.3), ribavirin (6.9%), metoprolol (6.0%), sertraline (5.8%), clopidogrel (5.6%), warfarin (5.1%), paroxetine (4.9%), voriconazole (4.9%) and tacrolimus (4.3%).

### Assessment of pharmacogenes and drug classes predominantly linked to DGI associated ADRs in Africa

**Figure 4:**
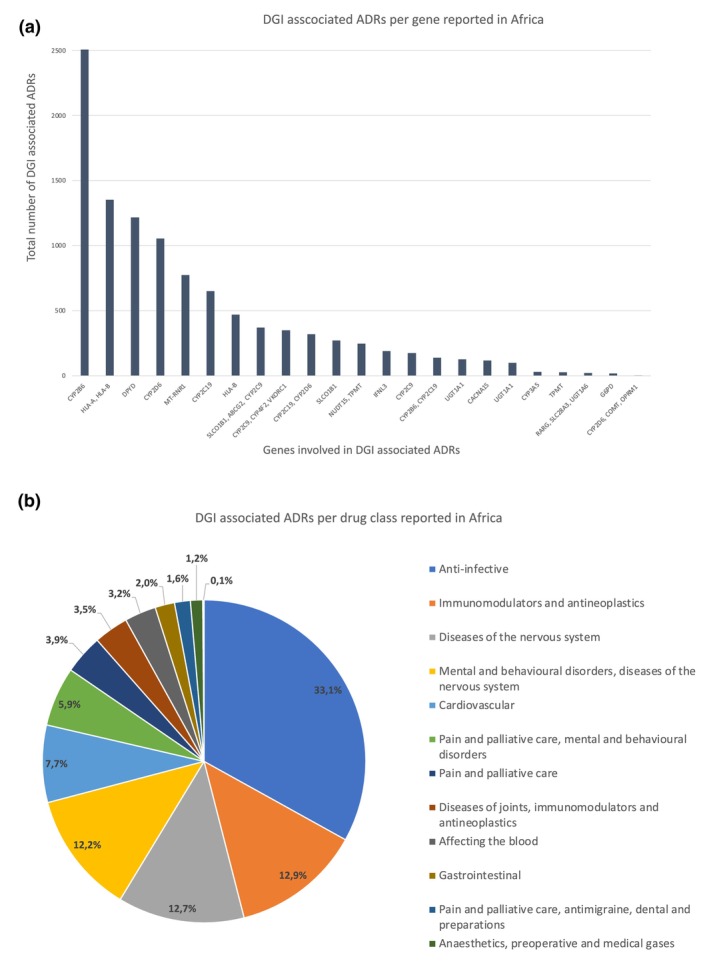
Sub‐analysis of the A. genes and B. drug classes involved in DGI associated ADRs in Africa as measured by the total number of potential DGI‐associated ADRs per gene or gene grouping and drug class or drug class grouping.

A sub‐analysis (**Figure** **4** **a**) indicates that the gene most associated with DGI associated ADRs reported for Africa is *CYP2B6*, potentially indicative of the extensive central nervous system adverse effects associated with efavirenz. Whilst DGI associated ADRs linked to *CYP2D6* alone rank this gene 4th (accounting for over 1000 DGI associated ADRs), in combination with *CYP219*, this gene grouping is associated with over 2000 reports placing it as a close second to the *HLA‐A* and *HLA‐B* gene grouping. The former is linked to several medicines within the mental health drug class, whilst the latter contributes almost exclusively to ADRs associated with hypersensitivity, including Steven Johnsons syndrome and other skin reactions. Of interest is the contribution of *MT‐MNR1* which is ranked 5th and is exclusively causative of hearing loss following administration of aminoglycoside antibiotics.

This contribution of medicines potentially associated with DGI‐associated ADRs covers a multitude of medicines including anti‐TB, ARVs, antipsychotics, antineoplastics, anticoagulants, anti‐seizure medication, anticonvulsants, anticancer, and antidepressants. When broken down into drug class (**Figure** **4** **b**), the class primarily associated with DGI‐associated ADRs is anti‐infective medicines, comprising 12 different MAPBs. Over 50% of the DGI‐associated ADRs can be attributed to MAPB reports classed within immunomodulators and antineoplastics, diseases of the nervous system in combination with mental and behavioral disorders, and diseases of the nervous system alone. VigiBase reports derived from cardiovascular medicines and medicines used for a combination of pain and palliative care as well as mental and behavioral disorders were associated with another 13.6% of DGI‐associated ADRs, highlighting key drug classes affected by PGx variations.

## DISCUSSION

Despite the many publications raising the importance and profile of ADRs in Africa, there is a lack of comprehensive pan‐African data analyses and a dearth of reports which take into account African genetic heterogeneity. This is particularly important given the sparse knowledge and/or appreciation of regional genetic diversity across the continent within the groups of stakeholders (healthcare workers and regulators) most able to implement and action the change required, given that such knowledge could shape disease risk and drug response outcomes.

In this study we were able to analyze ADRs submitted to the UMC over 10 years (2014 to 2023) with the objective of gathering a baseline understanding of those affecting geographical African populations taking into account those that have been associated with PGx variants in global guidelines which may have significant implications given Africa's well‐established genomic heterogeneity.

Of the over 500 medicines listed on the WHO EML list, we identified 56 which have PGx clinical guidelines from one or more international consortia, and referred to them as medicines associated with PGx biomarkers (MAPBs). Despite Africa being the second most populous continent, only 1% of global ADR reports associated with these 56 drugs are submitted from the African region. Whilst the manner in which VigiBase data is accrued enabled access to data from 47 African countries, the data cannot be assessed at a national level hence limiting the analysis of intercountry differences in reporting ADRs. It also further limited the ability to link regional or national frequency of genetic variants with reported ADRs. An interesting observation was that globally, these 56 drugs contribute to 12% of all ADRs reported in Africa, with the equivalent figure being 7% for the globe. This points to the possibility of Africa's diverse genetic heterogeneity contributing to higher incidences of ADRs in Africa and supports the need to include African populations in the clinical evaluation of medicines for safety and efficacy. Recently, Magavern et al. published an analysis of over 1 million UK reports attributed to 2000 different substances from 1963 to 2024, and revealed that 9% were associated with medications with PGx guidelines.^74^ Most of these were associated with *CYP2C19*, *CYP2D6*, and *SLCO1B1*, and nearly half were attributed to psychiatric medications, with a quarter linked to cardiovascular medications. Whilst only 26 of the medications used in this study overlapped with the 39 in Magavern et al., there are interesting comparisons that could tentatively be made between the two.

Unsurprisingly, our analysis revealed that the drug reports associated with the highest number of defined DGI associated ADRs submitted to VigiBase remain those used to treat infectious disease (especially efavirenz), in contrast to the Magavern study, which is likely due to the higher HIV disease burden and uptake of antiretrovirals. In addition, analysis of the VigiBase data revealed that over 40% of the efavirenz ADRs reported over the last decade on the African continent are well established in the literature as linked to variations in the *CYP2B6* gene.

Other medicines for which a significant majority of ADRs have been shown to be linked to PGx guidelines, include kanamycin (*MT‐RNR1*), haloperidol (*CYP2D6*), carbamazepine (*HLA‐A, HLA‐B*), and capecitabine (*DPYD*), ranging from 45 to 72% of ADRs for each MAPB. However, the collective gene grouping (after *CYP2B6*) constituting most DGI associated ADRs was that of *CYP2C19* and *CYP2D6*, similar to that reported by Magavern *et al*. 2025. The shared relative impact of *CYP2D6* and *CYP2C19* in DGI across both studies points to the importance of these two genes in any PGx guided precision medicine program. Warfarin also appears as a key medicine which should be considered, given increasing appreciation for the need for anticoagulants in sub‐Saharan Africa,^75^ associated with a high portion of DGI associated ADRs from medicines for cardiovascular disease.

We acknowledge that our analysis of VigiBase data is not an ideal source of data with which to define causality between PGx variants and specific DGI associated ADRs as has been published by the PREPARE study in Europe.^76^ In the former study, the association between the ADR, the drug in question and the genotype was assessed using the DWPG guidelines. In the absence of the genotype and the ability to apply the use of the Liverpool ADR Causality Assessment Tool,^77^ we are unable to assign specific causality. However, applying DWPG, CPIC, CPNDS and/or RNPGx guidelines to the dataset available in Africa, our findings provide evidence that could support the prioritization of drugs that require DGI bridging studies to improve their safe use in Africa. One such example is the high proportion of possible DGI associated ADRs in VigiBase reports from drugs well‐established in the literature to be linked to *MT‐MNR1*, variants which cause hearing loss following administration of aminoglycoside antibiotics.^78^ Given the *relative* ease of assessing hearing loss in children in a resource limited setting, and the significant impact of intervention, a bridging DGI study could be implemented on the continent. Other medicines for prioritization could be extracted from **Figure** **3** from among the top 10 with the highest DGI associated ADRs and inefficacy. The bridging studies could then be conducted across African populations with priority assigned to those with the highest associated disease burden and/or highest frequency of variants of causative genes.

It is of concern that so few ADRs are being reported for well‐established DGIs that lead to ineffective treatment. Over 30% of individuals of African ancestry harbor just one variant which will limit the ability of *CYP2D6* ^79^ to convert this to its bioactive form, endoxifen, yet less than 4% of the reports assessed here are associated with ineffective treatment. In addition, less than 5% of reports are associated with ineffective tacrolimus treatment despite this being associated with variants in *CYP3A5* present in 75% of African patients. The prevalence of these genetic variants reveals a significant gap in the reporting of ineffective treatment across the continent, reinforcing the need for pharmacovigilance approaches to detect signals of drug inefficacy required to be implemented in Africa.

This study serves as a valuable foundation with which to understand the landscape of DGI associated ADRs across Africa. However, there are limitations. Firstly, we were unable to create regional ‘maps’ of ADRs, which would be of significant value in informing regional stakeholders (researchers, healthcare professionals and regulators) with data that is driven by local disease burdens and local genetic diversity. This would also support the identification of countries with low reporting rates and ensure more equitable data interpretation. In addition, the definitions of DGI associated ADRs were restricted to those clinical phenotypes reported in the literature and may be conservative depending on the reporter. Whilst reports submitted to VigiBase have to meet a minimum standard of completeness, we acknowledge that there are uncertainties of causal links between medicines and ADRs, as well as a lack of patient exposure data which further limit the deductions that can be made from this dataset. The use of VigiBase data in the absence of genetic assessment precludes our analysis from showing direct causal links. In this sense, we cannot extricate the contributions of PGx alone, as several DGI associated ADRs may also be caused by polypharmacy, age, disease comorbidities and other factors which may lead to an over‐estimation of DGI associated ADRs. Collectively, therefore, the relative percentages of DGI associated ADRs should be treated as a high level interpretation of the available data, but can be used to arm our stakeholders with foundational evidence that DGI associated ADRs across the African continent are consistent with established known PGx associations. This may further lend itself to proactive and predictive pharmacovigilance decision‐making which, in combination with knowledge of local burden of diseases, drug procurement and allele frequencies of pharmacogenes, is much needed in Africa.

## FUNDING

The study was funded by the Bill and Melinda Gates Foundation (grant INV‐058365) for the development of the target policy profile framework for integrating Africa's genomic heterogeneity in drug discovery, development, and deployment.

## CONFLICTS OF INTEREST

The authors declared no competing interests for this work.

## AUTHOR CONTRIBUTIONS

J.S., T.A.M., M.N., D.T., G.A., C.M. wrote the manuscript; J.S., T.A.M., M.N., D.T., G.A., C.M. designed the research; J.S., M.N., T.M. performed the research; J.S., T.A.M., M.N., analyzed the data.

## Supporting information


**Table S1.** DGI associated ADRs.

**Table S2.** Analysis of total ADRs showing number of reports per region and % relative to each region per drug in the TALAGH ADR dataset.

**Table S3.** Analysis of DGI associated ADRs.
